# Prediction of Rivaroxaban-Rifampin Interaction After Major Orthopedic Surgery: Physiologically Based Pharmacokinetic Modeling and Simulation

**DOI:** 10.3389/fphar.2021.706781

**Published:** 2021-07-23

**Authors:** Rui-juan Xu, Tao Ling, Hong Tang, Wei-hong Ge, Qing Jiang

**Affiliations:** ^1^ Department of Pharmacy, Drum Tower Hospital Affiliated to Medical School of Nanjing University, Nanjing, China; ^2^ Department of Sports Medicine and Adult Reconstructive Surgery, Drum Tower Hospital Affiliated to Medical School of Nanjing University, Nanjing, China; ^3^ School of Basic Medicine and Clinical Pharmacy, China Pharmaceutical University, Nanjing, China; ^4^ Department of Analysis, Nanjing GQ Laboratories co., Ltd, Nanjing, China

**Keywords:** physiologically based pharmacokinetic, drug drug interaction, rivaroxaban, rifampin, prosthetic joint infection

## Abstract

Rivaroxaban is commonly used for the prophylaxis of venous thromboembolism (VTE) for patients undergoing major orthopedic surgery. Rivaroxaban is primarily eliminated by hepatic CYP450 metabolism and renal excretion. Rifampin is a commonly used antibiotic for prosthetic joint infections (PJI) and a potent inducer of CYP450 enzymes. Clinical data about drug-drug interactions of rivaroxaban and rifampin are limited. The present study is to describe DDI of rivaroxaban and rifampin in several prosthetic joint infections patients undergoing major orthopedic surgery. We retrospectively identified six patients concomitantly administered with rivaroxaban and rifampin between 2019 and 2020. Plasma samples of these patients with accurate sampling time were chosen from the biobank and plasma levels of rivaroxaban were measured at each time point. A physiologically based pharmacokinetic model for the rivaroxaban-rifampin interaction was developed to predict the optimal dosing regimen of rivaroxaban in the case of co-medication with rifampin. The model was validated by the observed plasma concentration of rivaroxaban from the above patients. From this model, it could be simulated that when rifampin starts or stops, gradually changing rivaroxaban dose during the first few days would elevate the efficacy and safety of rivaroxaban.

## Introduction

Rivaroxaban, a direct oral FXa inhibitor, is commonly used for the prophylaxis and treatment of venous thromboembolism (VTE), especially for patients undergoing major orthopedic surgery (Capodanno et al., 2012). Rivaroxaban undergoes complicated elimination process involving both hepatic metabolism and renal excretion. Hepatic metabolism includes cytochrome P450 (CYP 450) metabolism and hydrolysis in the liver. Renal excretion includes passive glomerular filtration (minor) and p-glycoprotein/breast cancer resistance protein (P-gp/BCRP) mediated active secretion (major). Although rivaroxaban is generally well tolerated, recent evidence suggests that certain drug-drug interactions (DDI) has the potential to affect the efficacy of rivaroxaban (Lang et al., 2009; Weinz et al., 2009; FDA, 2011; Gnoth et al., 2011).

Rifampin is an antibiotic used to treat several types of bacterial infections. Combined with other intravenous antibiotics, rifampin is the first-line choice for the treatment of Staphylococcal prosthetic joint infections (PJI) in patients undergoing total joint arthroplasty (Osmon et al., 2013). Rifampin is a well-known CYP 450 and P-gp inducer (Baneyx et al., 2014), both of which are important factors contributing to the elimination of rivaroxaban (Lang et al., 2009; Gnoth et al., 2011). Patients with PJI after major orthopedic surgery is involved with an increased risk of thromboembolic events, and in these circumstances rivaroxaban is generally prescribed for the long-term prophylaxis of VTE. However, there are case reports of patients suffering from embolism events as a result of decreased rivaroxaban exposure due to the co-medication of CYP/P-gp inducer (rifampin and phenytoin), and the drug labeling of rivaroxaban does not recommend the concomitant use with rifampin (FDA, 2011; Altena et al., 2014; Becerra et al., 2017). In clinical practice, medical practitioners are often left to juggle anticoagulation and antibiotic treatment decisions. Thus the concomitant use of rivaroxaban and rifampin may not be completely avoided and optimization of dosing regimen is needed in these cases.

So far, the pharmacokinetic study of rivaroxaban combined with rifampin is limited. The aim of this report is to describe DDI of rivaroxaban and rifampin in several PJI patients undergoing major orthopedic surgery. Since physiologically based pharmacokinetic (PBPK) modeling can be used for prediction of DDI and dosing regimen optimization (Min and Bae, 2017), we further quantify the time course of rivaroxaban concentrations with or without rifampin utilizing PBPK model based on plasma samples taken from the present patients. Most importantly, we aim to provide dosage insights and recommendations to better serve patients who require rivaroxaban combined with rifampin.

## Method

### Pharmacokinetics of Rivaroxaban-Rifampin Interaction in Patients With Prosthetic Joint Infections

**FIGURE 1:**
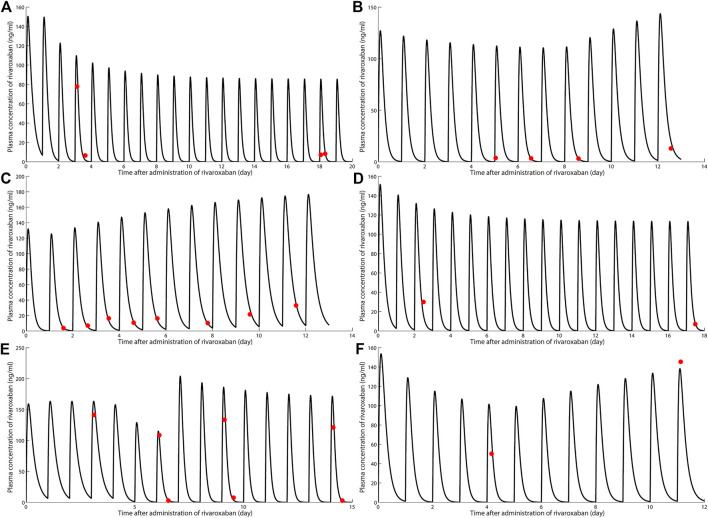
Simulation of pharmacokinetic profiles of rivaroxaban with or without rifampin in different dosing regimens. Prediction of the concentration-time courses of rivaroxaban in the present patient cases **(A–F)**. The line represents the prediction and the dot presents the observed value.

Six PJI patients with concomitant administration of rivaroxaban and rifampin between 2019 and 2020 were retrospectively identified. Liver enzymes, and coagulation assays of all patients were reported within normal ranges. Caprini risk assessment (Gould et al., 2012) showed that the selected patients were at high risk of VTE and rivaroxaban was chosen for VTE prophylaxis. Treatment with rifampin was determined by laboratory cultivation and drug sensitivity test, which showed the purulent synovial fluids from all the patients had staphylococcus infections that were sensitive to rifampin treatment according to the guideline (Osmon et al., 2013). The orthopedist who prescribed rifampin may not have been aware of the potential DDI between rivaroxaban and rifampin. Details of patients’ physical condition and dosing regimens of rivaroxaban and rifampin are listed in Table 1. Plasma samples from these patients with records of sampling times were chosen from the hospital biobank. Rivaroxaban plasma concentrations were determined using a validated and selective chromatographic assay with mass spectrometric detection (LC-MS/MS) (Rohde, 2008). The observed rivaroxaban plasma concentrations at different time points in each patient are shown in Figure 1. Informed consent was obtained from all patients, and the study was approved by the Ethical Committee of Drum Tower Hospital Affiliated to Medical School of Nanjing University (Nanjing, China).

**TABLE 1:** Patients’ physical condition and dosing regimens of rivaroxaban and rifampin.

case NO.	Age	Weight	Sex	Creatinine clearance (ml/min)	Drug administration
1	64	60.3	Male	118	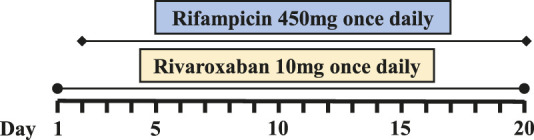
2	70	65	Female	70	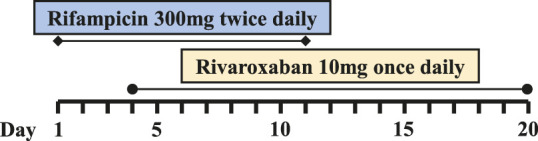
3	70	61.5	Male	110	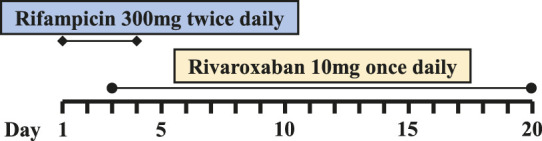
4	71	67	Male	52	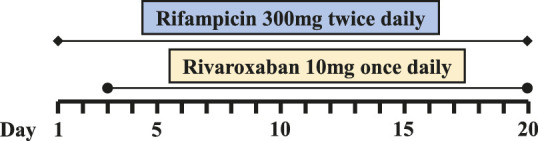
5	61	64.5	Female	106	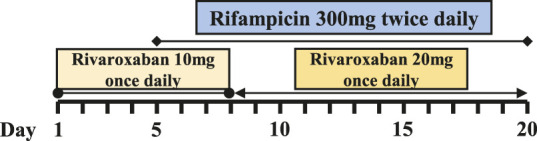
6	36	60	Female	129	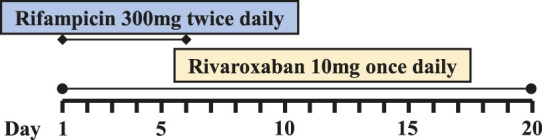

### Physiologically Based Pharmacokinetic Modeling of the Rivaroxaban-Rifampin Drug Drug Interaction

A rivaroxaban-rifampin interaction PBPK model was developed to predict the optimal dose of rivaroxaban with the co-medication of rifampin. The whole-body PBPK model of rivaroxaban was previously published (Xu et al., 2018), and the DDI impact of rifampin on rivaroxaban was modeled by including the induction effects of rifampin on CYP3A and P-gp.

A one-compartment pharmacokinetic model with 1st order absorption and elimination was constructed for the rifampin component of the rivaroxaban-rifampin interaction model. Considering that rifampin exposure was significantly decreased after repeated dosing, which is indicative of self-induction in its hepatic metabolizing enzyme, a self-induction mechanism was also integrated in the model (Peloquin et al., 1997; van Ingen et al., 2011). The integrated rivaroxaban-rifampin model allows simulation of the impact of rifampin on renal and hepatic clearance of rivaroxaban.

The effect of the inducer on the kinetics of hepatic or renal compartment is described as follows (Baneyx et al., 2014):

$$\frac{dE_{t,i}}{dt}=K_{\deg}\times E_{0}\times \frac{E_{\max}\times f_{u,inducer,i}\times C_{inducer,i}}{EC_{50}\times f_{u,hep}+f_{u,inducer,i}\times C_{inducer,i}}+K_{\deg}\times \left(E_{0}-E_{t,i}\right)\left(\text{i }=\text{ liver or renal}\right)$$ (1)

$$CL_{CYP,induce}=CL_{CYP}\times E_{t,liver}$$ (2)

$$CL_{tubular,P-gp,induce}=CL_{tubular,P-gp}\times E_{t,renal}$$ (3)

Estimation of the total clearance of rifampin with self-induction is described as follows:

$$CL_{t,rifampin,ind}=CL_{R,rifampin}+f_{m,CYP3A4}\times E_{t,liver}\times \left(CL_{t,rifampin}-CL_{R,rifampin}\right)+\left(1-f_{m,CYP3A4}\right)\times \left(CL_{t,rifampin}-CL_{R,rifampin}\right)$$ (4)

Where E_t_ and E_0_ are the relative CYP3A4 activity at time t and time 0, respectively. C_inducer, liver_ is the inducer levels in the liver and f_u,inducer, liver_ is the free fraction of inducer in the liver. In the absence of induction, the relative CYP3A4 activity is equal to E_0_ and set to 1. EC_50_ represents the inducer concentration required to reach half of the maximum enzyme activity (E_max_). EC_50_ is corrected by the free fraction of inducer in hepatocytes (f_u,hep_) of 0.42 during incubation with human primary hepatocytes. K_deg_ is the degradation rates of CYP3A4 enzyme in the liver (Baneyx et al., 2014). As previously reported (Qian et al., 2019), since expressions of both P-gp and CYP3A4 were regulated by pregnane xenobiotic receptor, we assumed that induction parameters of P-gp by rifampin were similar to those of CYP3A4 and that expression of P-gp during rifampin treatment was also estimated by Eq. 1. CL_t,rifampin_ is the total clearance of a single dose of rifampin (without self-induction). CL_t,rifampin,ind_ is the total clearance of rifampin with self-induction. f_m,CYP3A4_ is the fraction of contribution to intrinsic hepatic clearance of rifampin from CYP3A4 enzymes. CL_R,rifampin_ is the renal clearance of rifampin. The pharmacokinetic parameters of rifampin were obtained from previous pharmacokinetic studies and shown in Table 2 (Peloquin et al., 1997; Templeton et al., 2011; Xu et al., 2011; Baneyx et al., 2014).

**TABLE 2:** Parameters used in the induction model.

Parameters	Values	Units
E_0_	1.0 Baneyx et al. (2014)	Fold
E_max_	9.0 Templeton et al. (2011)	Fold
EC_50_	0.8 Templeton et al. (2011)	μM
K_deg_	0.0096 Baneyx et al. (2014)	h^−1^
K_p_(liver)	0.27 Baneyx et al. (2014)	
f_u_(liver)	60.9 Baneyx et al. (2014)	%
First-order absorption rate constant: K_a_	0.58 Peloquin et al. (1997)	h^−1^
Volume of distribution: V	0.33 Xu et al. (2011)	L/kg
Total clearance: CL_t_	7.4 Baneyx et al. (2014)	L/h
Renal clearance: CL_R_	1.5 Baneyx et al. (2014)	L/h
Fraction of CYP3A4: f_m_	0.2 Baneyx et al. (2014)	

### Simulation Design

The model described above was implemented in MATLAB software (The MathWorks, Inc., Natick, MA, United States). The model performance was evaluated by comparing simulated rivaroxaban profiles with the observed data in the aforementioned patients. The simulation was conducted in a way mimicking the real-life experience, i.e., by using the patients’ baseline characteristic and dosing history of combination drugs as shown in Table 1.

### Simulation Software

All simulations were performed using MATLAB (the MathWorks Inc., Natick, MA, United States). The PBPK model was constructed as a set of ODEs, the integration of which was performed using the fourth order Runge-Kutta method.

## Results

### Simulations

**FIGURE 2:**
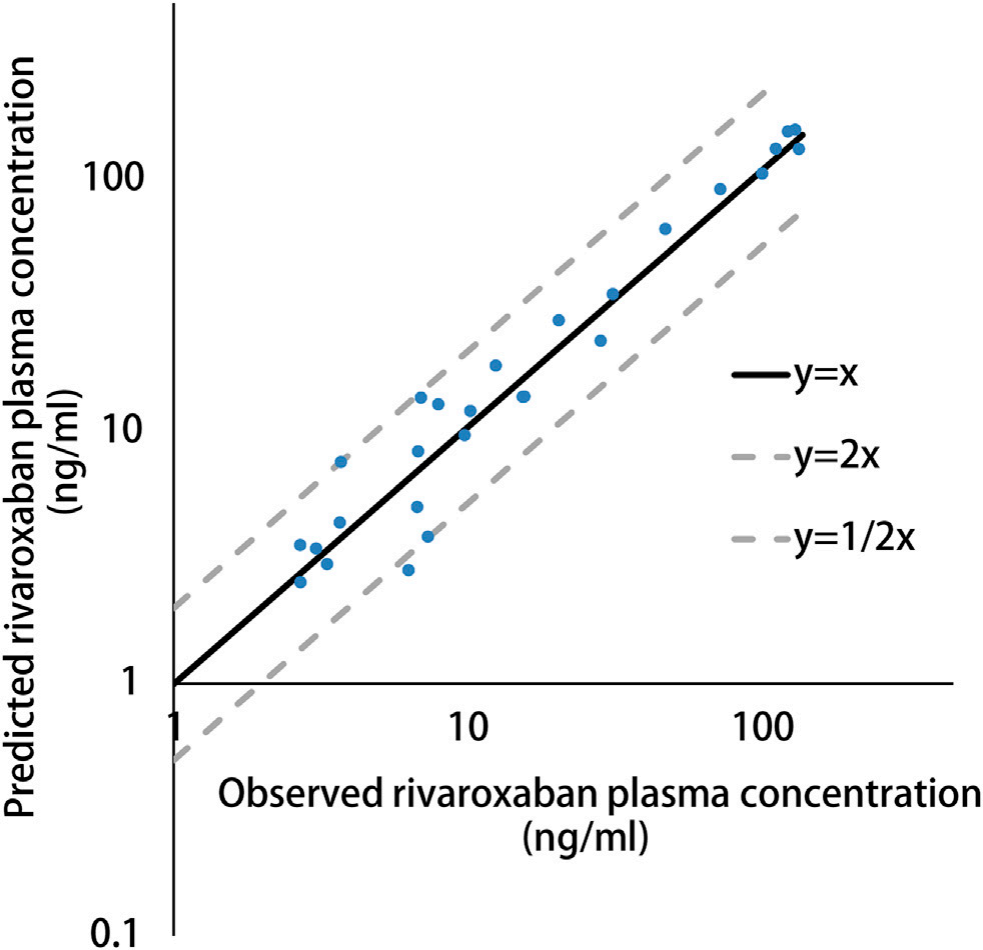
Predicted versus observed plasma concentration of rivaroxaban in present patient cases.

The simulated results are shown in Figure 1 and Figure 2, in which a good agreement of the observed PK concentrations and simulated PK profiles is observed and almost all the fold errors (If the observed value is greater than the predicted value, fold-error = observed/predicted; If observed value is less than the predicted value, fold-error = predicted/observed) were less than two. Consistent with the time-dependent CYP3A4 induction by rifampin, the exposure of rivaroxaban gradually decreased and finally reached a steady state. This indicates that the revised PBPK model is suitable for the simulation of dosing regimens of rivaroxaban when co-administered with rifampin.

### Recommendation of the Dosage Modification

Based on previous cases study, we want to know the appropriate dosing regimen if rivaroxaban was the choice for patients combined with rifampin. As specific concentration cut-offs associated with a risk of bleeding or thrombosis are currently not established, the plasma concentration range (5th-95th percentile) of the reported pharmacokinetic studies was used as references in the current analysis (Bernier et al., 2020). For patients undergoing total hip arthroplasty receiving rivaroxaban 10 mg qd, 5th-95th percentile of maximum concentration (C_max_) and area under the concentration-time curve (AUC) is 91–196 ng/ml and 771.5–2,118.2 ng h/l, respectively (Mueck et al., 2008). In the present study, AUC was used as an exposure variable for both efficacy and safety, whereas C_max_ was used as an exposure variable for safety. Thus, AUC lower than 771 was considered with a higher risk of thrombosis and C_max_ higher than 196 ng/ml or AUC higher than 2,118 ng h/l was considered with a higher risk of bleeding. Consequently, several scenarios were simulated for the first patient as the example to find an optimized dosing regimen.

**FIGURE 3:**
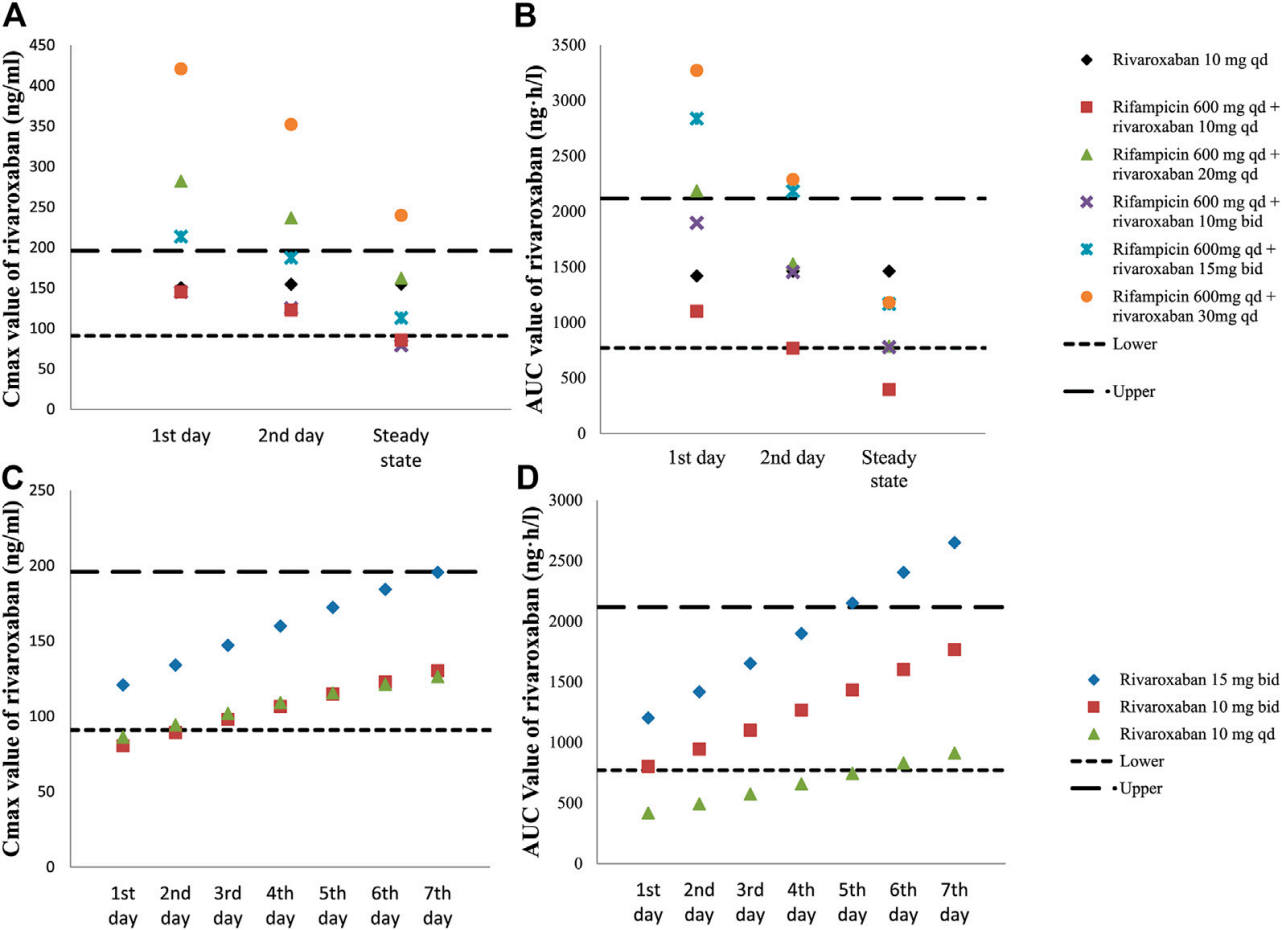
Simulated exposure of rivaroxaban with or without rifampin. C_max_ **(A)** and AUC **(B)** values on the first day, second day and steady state in different dosing regimens of rivaroxaban-rifampin co-medication. C_max_ **(C)** and AUC **(D)** values from the first day to the 7th day after withdrawal of rifampin in different dosing regimens.

Several dosing regimens were simulated assuming rifampin was dosed at the same time as rivaroxaban, and the results are illustrated in Figures 3A,B, which show both C_max_ and AUC value on the first day (C_max,1st_ and AUC_1st_), second day (C_max,2nd_ and AUC_2nd_) and at the steady state (C_max,ss_ and AUC_ss_). As can be seen in Figures 3A,B, 15 mg bid is the only cohort with both C_max,ss_ and AUC_ss_ fall within the target exposure range. However, C_max_ and AUC of this cohort on the first day were higher than the upper bound of the target exposure window. This can be potentially mitigated by reducing the dose on the first day to 10 mg, ie, the patient will receive 10 mg qd on the first day and 15 mg bid starting on the 2nd day, which will have all the C_max_ and AUC value fall in the target exposure window (shown as Figure 3).

Simulations were also conducted to investigate the adjustment of rivaroxaban doses after withdraw of rifampin under steady state. As shown in Figures 3C,D, the gradual decrease in rivaroxaban clearance is associated with increased C_max_ and AUC after rifampin was withdrawn. Since this is a gradual process, AUC of rivaroxaban was predicted to be lower than 771 ng h/ml during the first 5 days if rivaroxaban was changed to 10 mg qd immediately after rifampin withdraw, which might be suggestive of increased thrombotic risk. On the other hand, AUC of rivaroxaban was predicted to be higher than the upper bound 5 days after rifampin withdraw if the dosing regimen of rivaroxaban was maintained at 15 mg bid, which might be suggestive of increased bleeding risk (shown as Figure 3D). Therefore, in order to make all the AUC and C_max_ values fall into the target window, the dose adjustment after the withdrawal of rifampin should be gradual, and during the first 7 days should be in the following order: 15, 10, 10 mg qd.

## Discussion

Rivaroxaban belongs to the family of direct-acting oral anticoagulants (DOACs) that does not need a routine laboratory testing. However, the inductive interaction should be considered when treating PJI combined with thrombosis prophylaxis because rifampin affects the dose and dosing frequency of rivaroxaban required. The simulations showed that the rivaroxaban dose should not be changed immediately after the start or stop of rifampin. While it is generally difficult to know how to optimize the dosing regimen of rivaroxaban when combined with rifampin, our simulations show that when rifampin is added or removed from patient’s therapy, a gradual change in rivaroxaban dose would potentially increase the efficacy and safety profiles of rivaroxaban.

Although rivaroxaban is not recommended to be used together with rifampin due to their DDI risk, the present study found a reasonable way to optimize this combination therapy, and rivaroxaban might become an option for anticoagulants when rifampin is being used.

The present model was expanded from our previous PBPK model for rivaroxaban which was constructed from data from literature. The previous model could be used to predict rivaroxaban pharmacokinetics in patients with renal and hepatic dysfunction and inhibitory drug drug interactions. In the present study, a time-dependent inductive DDI model for rifampin was added into the basic rivaroxaban model to predict the effect of rifampin on rivaroxaban pharmacokinetics. The final model was subsequently evaluated by the PK data in six patients. Simulations were conducted to optimize dosing regimen in the aim of improving the efficacy and safety of this combination. Although there are several markers indicating the effect of rivaroxaban, such as APTT and PT, we chose serum concentrations to aid in evaluating the efficacy and safety which can sensitively reflect the change caused by DDI.

Trough concentration (C_trough_), average concentration and AUC are usually used as exposure variables for both efficacy and safety, while maximum concentration is used as an exposure variable for safety. In the present study, C_trough_ was not considered as the parameter evaluating the efficacy or safety. For oral administration of rivaroxaban 10 mg qd, the 5/95 percentile range of C_trough_ is 1.3–37.6 ng/ml with a quite large variability and a quite low lower bound which indicated a larger detection error at lower concentration. Therefore, AUC was considered as a better parameter for evaluation of the efficacy or safety of rivaroxaban in the present study, and the close correlation between AUC and C_trough_ made this less of a concern.

There are multiple benefits to use the model-informed approach when searching for the optimal dosing regimen in DDI scenarios. Previous pharmacokinetic observations could be taken into account for model development and predictions. The developed model can predict future exposure resulting from a proposed adjusted dose, which could serve as our best guess of the expected exposure before any trial data become available. Moreover, individual characteristics can be further included in the model, which provides more informed individual predictions. The major limitation of this work is the limited data we have to validate the model, and such a small sample size also made it impossible to accurately characterize the variability among patients. Additionally, plasma rivaroxaban concentration range (5th-95th percentile) from pharmacokinetic studies was set as the alternative cut-offs since no specific cut-offs was established for the risk of bleeding or thrombosis. This approach may introduce some inaccuracy and make the final prediction deviate from the actual situation. Finally, prediction results from the present model should be re-evaluated in the clinical practice.

## Conclusion

The PBPK model used in the current study was able to predict pharmacokinetic profiles of rivaroxaban in the absence and presence of rifampin. Simulation indicated that gradual adjustment of rivaroxaban dose during the first few days after initiation or termination of rifampin is warranted to increase the efficacy and safety profiles of rivaroxaban.

## Data Availability

The original contributions presented in the study are included in the article/Supplementary Material, further inquiries can be directed to the corresponding authors.
